# The association between common mental disorders and tuberculosis: a case–control study from Guinea-Bissau

**DOI:** 10.1017/neu.2024.5

**Published:** 2024-02-08

**Authors:** Lena Larson, Grethe Lemvik, Frauke Rudolf, Victor Francisco Gomes, Andreas Schröder, Christian Wejse

**Affiliations:** 1 Bandim Health Project, Bissau, Guinea-Bissau; 2 Department of Infectious Diseases, Aarhus University Hospital, Aarhus, Denmark; 3 Research Clinic for Functional Disorders and Psychosomatics, Aarhus University Hospital, Aarhus, Denmark; 4 GloHAU, Centre for Global Health, School of Public HealthAarhus University, Aarhus, Denmark

**Keywords:** Pulmonal tuberculosis, depression, anxiety, communicable disease, neuropsychological screening tools

## Abstract

**Objective:**

The aim of the study was to explore the association between tuberculosis (TB) and common mental disorders (CMD), in an area with high prevalence of TB.

**Methods:**

We performed a case–control study of TB patients and unmatched healthy controls, from a demographic surveillance site in Guinea-Bissau. Screening for CMD was performed once for controls and at inclusion and follow-up for TB patients. Kessler 10 (K-10) and a brief version of Hopkins Symptom Checklist 25 (SCL-8d) were used as screening instruments.

**Results:**

571 controls were interviewed and 416 interviews were performed for 215 TB cases. Estimated CMD prevalence at the time of diagnosis of TB was 33.6 % (SCL-8d) and 46.2 % (K-10), compared with 6.8 % (SCL-8d) and 6.7 % (K-10) among controls; adjusted OR 7.18 (95 % CI 4.07 to 12.67) and 14.52 (95 % CI 8.15 to 25.84), respectively. No significant difference in CMD prevalence rates was observed between TB patients, after 6 months of treatment, and controls.

**Conclusion:**

Psychological distress and common mental disorders were more prevalent among TB patients at the time of diagnosis compared with the background population, but after completion of TB treatment no increased prevalence of psychological distress was found.

Significant outcomes

- TB patients had higher occurrence of CMD at the time of diagnosis compared with the background population.
- CMD prevalence decreased during treatment.
- Applied screening tools, K-10 and SCL-8d, estimated various prevalence of CMD.

Limitations

- The number of controls eligible for CMD screening was 571 out of a randomised sample of 1500, and the distribution of sex was unequal between TB cases and controls.
- We were not able to collect follow-up data for each TB patient at each time point of follow-up.
- Although controls were screened for TB, there may be controls with TB in our cohort.

## Background

Tuberculosis (TB) remains a major global health challenge. WHO has estimated 10 million new TB cases and 1.5 million deaths resulting from TB in 2020 (WHO 2021). It is estimated that mental disorder may be attributed to 418 million disability-adjusted life years (DALYs) in 2019 (16% of global DALYs) (Arias *et al*, 2022) Three-quarters occur in low-and middle-income countries (LMIC), where there is a substantial gap between disease burden and access to mental health care (Mental Health Atlas, 2020).

There has been a rising focus on the interplay between psychiatric comorbidities and TB (Sweetland *et al*., 2017). Depression may cause immunosuppression, lower compliance and undernutrition, leading to increased of mortality and community transmission (Pachi *et al*., 2013; Ruiz-Grosso *et al*., 2020), whereas chronic inflammation in TB patients and some anti-tuberculosis medication may increase the risk of psychiatric disease (Doherty *et al*., 2013).

Common mental disorders (CMD) is a term which contains anxiety and depression (Henningsen *et al*., 2003). CMD is a useful concept due to the high degree of comorbidity and similarity of epidemiological profiles (Patel *et al*., 2009). Several studies have investigated the association between CMD and TB, but the results are diverse with observed prevalence rates, ranging between 28% and 80 % (Issa *et al*., 2009; Deribew *et al*., 2010; Peltzer *et al*., 2012; van den Heuvel *et al*., 2013; de Araújo *et al*., 2014; Hayward *et al*., 2022). Comparability in-between studies may be complicated due to methodological differences, and very few studies have reported CMD prevalence in TB patients compared with the background population (Aghanwa and Erhabor, 1998; de Araújo *et al*., 2014; Oh *et al*., 2017; Cheng *et al*., 2017).

We aimed to describe the relationship between CMD and TB further, in an area with a high prevalence of TB. Our primary outcomes were the association between prevalence of CMD and pulmonary TB at the time of diagnosis, during treatment and once treatment was completed. Data were collected in 2013–2014 from a continuing cohort of TB patients and from a randomised sample of controls obtained from a surveillance database, in Guinea-Bissau.

## Materials and methods

The Bandim Health Project has been a Health and Demographic Surveillance Site, located in Bissau, since 1978 (Gomes *et al*., 2011). The study area consists of six suburban areas, and the population is followed by regular demographic surveys. All individuals in the area are registered with their id number, age, sex, ethnic group and socio-economic data. Guinea-Bissau has an incidence of pulmonary TB of 279/100 000 person-years (Lemvik *et al*., 2014). There are no previous data of CMD prevalence in this population.

### Inclusion of cases and controls

A randomised sample of unmatched controls, ≥18 years, was obtained from surveillance data from the study area. The randomised sample of controls will be referred to as background population throughout the text. Controls were visited and interviewed at home by a trained field assistant and were paid three visits before the person was considered as non-eligible, as described by Virenfeldt (Virenfeldt *et al*., 2014). Standardised questionnaires were used to obtain information on sociodemographic characteristics and symptoms of TB and CMD. Controls with ≥1 symptoms of pulmonary TB, such as cough, haemoptysis, dyspnoea, chest pain and night sweats, were investigated for TB as described by Rudolf et al (Bjerregaard-Andersen *et al*., 2010; Porskrog *et al*., 2011; Rudolf *et al*., 2014). Controls diagnosed with TB were excluded.

Since 1996, a surveillance system has detected all patients diagnosed with and treated for TB in the study area. The field assistant identified TB patient during daily visits at the TB treatment facilities. Patients’ diagnosis has been made according to WHO criteria (Gomes *et al.*, 2013). TB cases were obtained from this continuous TB cohort, if diagnosed with pulmonary TB and ≥18 years. (Rudolf *et al*., 2014). All TB patients were treated with ethambutol, isoniazid, rifampicin, and pyrazinamide for two months, followed by isoniazid and ethambutol for four months. When enrolled in the TB cohort, patients were clinically examined, interviewed regarding symptoms and background information, and tested for HIV, as described elsewhere (Rudolf *et al*., 2014). Patients were followed up at two, four and six months to assess treatment outcome. Screening for CMD was performed when patients visited the healthcare centre at inclusion or at follow-up in the TB cohort. Due to the time frame of the study and the fact that some TB patients skipped controls, we were not able to screen each patient at all time points. In order to screen as many patients as possible at each time point, all TB patients coming to either inclusion or follow-up in the TB cohort were screened, whether they had been screened before or not. The TB patients were divided into four groups based on time point of screening; at inclusion, at two months, at four months and at six months. Some patients contributed to data in several groups, whereas some patients contributed to data in only one group. Our aim was to compare CMD among TB patients at the time of diagnosis, during treatment and once treatment was completed with a sample of randomly selected controls representing the background population; thus, TB patients were possibly screened at several time points during the inclusion period and controls only once. Screening for CMD in controls was performed continuously, parallel with the screening for CMD among TB patients. There was no available treatment for psychiatric disorders in Guinea-Bissau at the time of the study, and we were not able to offer treatment for patients screened positive for CMD.

All questionnaires, including CMD screening tools, were in written Portuguese and translated by the field assistant into the local language Creole. All screenings for CMD were performed by only one field assistant, and all interviews were regularly supervised by the primary investigator (LL).

### Screening tools for CMD

We chose two screening tools in order to screen for CMD, designed to detect symptoms of psychological and emotional distress. Psychological distress (PD) will be used throughout the text to address psychological and emotional stress and the risk of having CMD.

The Kessler 10 scale (K-10) contains 10 items regarding depression, tiredness, nervousness, restlessness, hopelessness and worthlessness, during 30 days prior to the survey. Each question is rated on a 5-point Likert scale (1–5), ranging from ‘never’ to ‘all the time’. K-10 has been validated in low-resource settings, with various recommendations of cut-off values (Stolk *et al*., 2014). Andrews et al estimated a sensitivity of 0.94 and a specificity of 0.63 for CMD at a cut-off at ≥14 (Andrews and Slade, 2001). In order to identify persons at risk of CMD, a cut-off of ≥14 was chosen to estimate prevalence of PD.

SCL-8 is an 8-item abbreviated version of Hopkins Symptom Check List (HSCL-25) containing questions concerning nervousness, fear, spells of panic, feeling blue, worrying, hopelessness, feeling everything is an effort and worthlessness (Fink *et al*., 2004a). It has been validated in a population with medical patients, psychiatric patients and in the background population (Fink *et al.*, 1995; 2004b). Each question has five response categories (0–4), ranging from ‘not at all’ to ‘extremely’, 30 days prior to the interview. In a validation study, items were dichotomised, in a way that the categories ‘not at all’ and ‘a little bit’ were categorised as negative responses and ‘moderately’, ‘quite a bit’ and ‘extremely’ as positive responses (Fink *et al*., 2004b). A cut-off of ≥1 on the dichotomised SCL-8 scale was used to identify cases at risk of having CMD, with a sensitivity of 0.73 and a specificity of 0.61 for a medical setting (Fink *et al*., 2004b). Described screening tool is referred to as SCL-8 dichotomised (SCL-8d) and is used to estimate PD prevalence.

### Data handling

**Figure 1.**
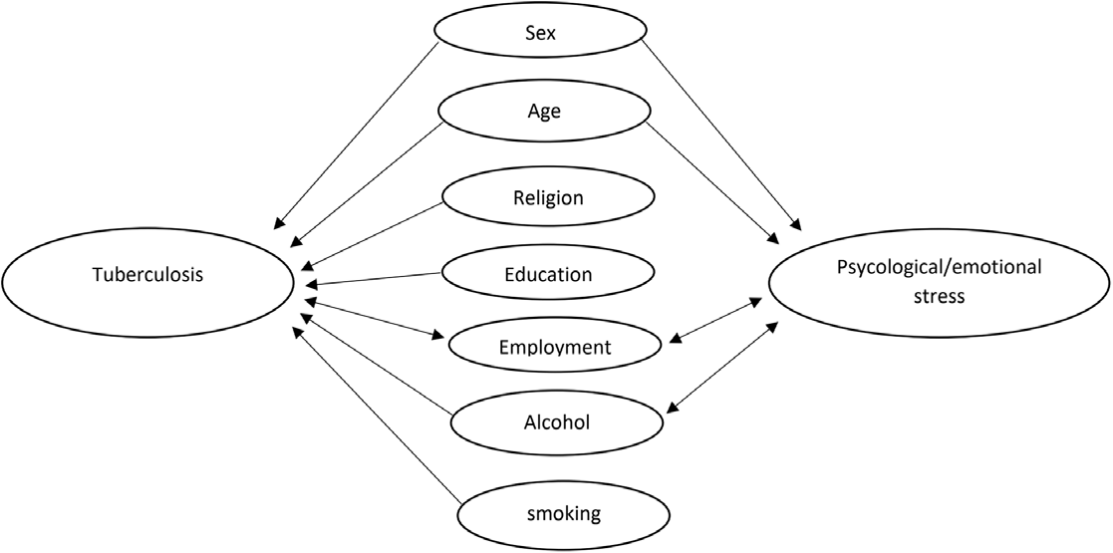
Directed acyclic graph to evaluate connections between variables.

Data were entered using dBase V software and access 2007, and transferred to STATA (version 11) for analysis. To assess statistical differences in demographic characteristics at baseline, Pearson’s X^2^-test was used for categorical variables, and ANOVA table test for continuous variables. Sex, civil status, ethnicity, religion, education, employment, smoking, alcohol and civil state were entered as categorical variables, and age was entered as a continuous variable. Statistical differences in sex and age were assessed between eligible and non-eligible controls. Mean score for PD was calculated for K-10 and SCL-8, and prevalence of PD was estimated by using cut-off scores for K-10 and SCL-8d. Difference in PD prevalence between TB case and controls was estimated by odds ratio (OR), with 95% confidence interval (CI), using logistic regression. We observed significant difference in sociodemographic characteristics for sex, age, religion, education, employment, alcohol and smoking, but due to the setup of the study we were not able to define all of them as pre-exposure covariates. To assess statistical differences in sociodemographic characteristics with k-10 and SCL-8 outcome, crude logistic regression and adjusted OR were calculated for each of the sociodemographic characteristics at baseline. Sex, employment, alcohol and age, each respectively, changed the estimate ≥10%. A directed acyclic graph was drawn (Fig. 1) based on analyses and evidence from known risk factors for tuberculosis and common mental disorder (Bitew, 2014; Horton *et al*., 2016; Neyrolles and Quintana-Murci, 2009; 2016; Silva *et al*, 2018; Steel *et al*., 2014). Religion, education and smoking, only affected one of the main variables (TB) and were defined as potential mediators. To avoid unstable estimates, they were not included in the final model. Employment status was defined as a potential collider, due to the assumption that it may be affected by both main variables TB and PD. To avoid the risk of introducing bias, it was not included in the multivariate model. Sex, alcohol and age were assumed to affect both TB and PD and were included in the multivariable regression model as potential confounders. Cronbach’s alpha was used to evaluate internal consistency.

### Ethics

The project has been reviewed and approved by the National Ethics Committee in Guinea-Bissau (NoRef0406/CNES/INASA/2013). Before inclusion, TB patients were verbally informed in the common language Creole and written Portuguese. Informed consent was obtained by signature or by a fingerprint if illiterate. The authors assert that all procedures contributing to this work comply with the ethical standards of the relevant national and institutional committees on human experimentation and with the Helsinki Declaration of 1975, as revised in 2008.

## Results

**Figure 2.**
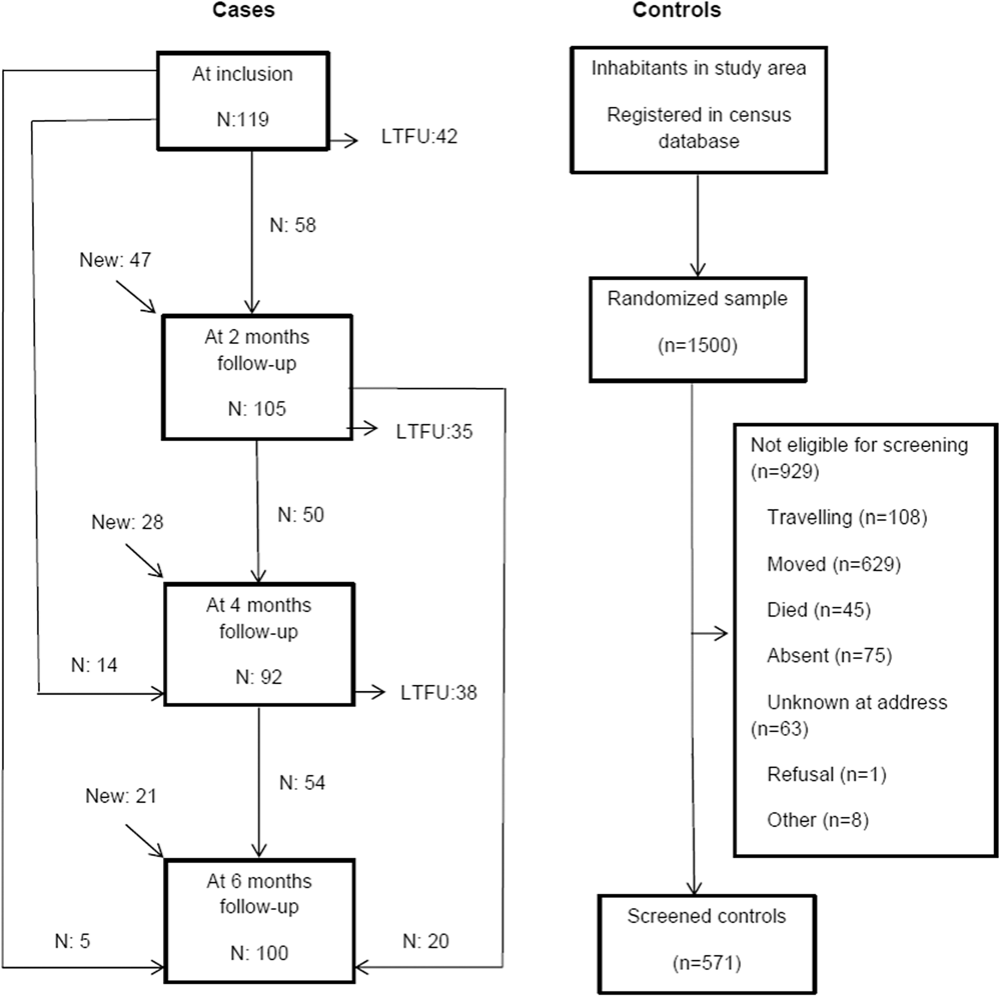
Flow chart of cases, with number of TB patients available for PD screening at each time point (LTFU: Lost To Follow Up) and flow chart of controls.

From April 2013 to June 2014, 571 interviews were performed for controls and 416 for TB cases. A total number of 215 TB patients were interviewed; 119 at inclusion, 105 at two months, 92 at four months and 100 at six months (Fig. 2).

Out of the randomised sample of unmatched controls of 1500 people, 571 controls were eligible for screening (Fig. 2). The most frequent cause for being non-eligible was relocation and travelling. Non-eligible controls were more likely to be male, but no significant difference was observed in mean age between eligible and non-eligible controls (Data not shown).

### Characteristics of cases and controls

Significant differences between cases at the different time points and controls were observed for several of the background characteristics (Table 1). At all time points, cases were more likely to be male and being smokers.

**Table 1.** Sociodemographic characteristics of cases at each time point in the TB cohort and randomised controls from background population

	Cases‡												
	At inclusion			At 2 months			At 4 months			At 6 months			Controls ‡
	N = 119*	P-value*		N = 105*	P-value*		N = 92*	P-value*		N = 100*	P-value*		N = 571*
**Sex *n* (%)**	–	>0.001		–	>0.001		–	>0.001		–	>0.001		–
Male	80 (67)	–		67 (64)	–		51 (55)	–		58 (58)	–		221 (39)
**Age**	–	0.535		–	0.292		–	0.270		–	0.950		–
*Mean (SD)*	34.7 (14.7)	–		32.3 (12.9)	–		35.5 (13.3)	–		33.9 (12.9)	–		33.8 (13.9)
**Ethnicity *n (%)***	–	0.792		–	0.414		–	0.714		–	0.050		–
Balanta	12 (10)	–		10 (10)	–		8 (9)	–		9 (9)	–		34 (6)
Fula	17 (14)	–		12 (11)	–		8 (9)	–		6 (6)	–		81 (14)
Mancanja	11 (9)	–		11 (10)	–		10 (11)	–		5 (5)	–		66 (12)
Mandinga	10 (8)	–		5 (5)	–		10 (11)	–		9 (9)	–		49 (9)
Manjaco	13 (11)	–		12 (11)	–		13 (14)	–		19 (19)	–		69 (12)
Pepel	38 (32)	–		33 (31)	–		28 (30)	–		35 (35)	–		187 (33)
Other	18 (15)	–		22 (21)	–		15 (16)	–		17 (17)	–		85 (15)
**Religion *n (%)***	–	>0.001		–	>0.001		–	>0.001		–	0.022		–
Iran (local religion)	33 (28)	–		24 (23)	–		22 (24)	–		22 (22)	–		107 (19)
Catholic	40 (34)	–		37 (35)	–		41 (45)	–		45 (45)	–		231 (40)
Protestant	9 (8)	–		11 (10)	–		3 (3)	–		7 (7)	–		62 (11)
Muslim	32 (27)	–		27 (26)	–		22 (24)	–		23 (23)	–		169 (30)
Other	5 (4)	–		6 (6)	–		4 (4)	–		3 (3)	–		2 (0,4)
**Education *n (%)***	–	0.202		–	0.037		–	0.315		–	0.698		–
0 years	30 (26)	–		17 (17)	–		21 (23)	–		21 (21)	–		106 (19)
1–6 years	24 (21)	–		13 (13)	–		17 (19)	–		17 (17)	–		122 (21)
7–9 years	28 (24)	–		34 (33)	–		25 (27)	–		20 (20)	–		123 (22)
>10 years	35 (30)	–		39 (38)	–		28 (31)	–		42 (42)	–		219 (38)
**Employment *n (%)***	–	>0.001		–	>0.001		–	>0.001		–	>0.001		–
Employed	78 (66)	–		59 (56)	–		55 (60)	–		58 (58)	–		174 (31)
**Smoking *n (%)***	–	0.006		–	>0.001		–	>0.001		–	>0.001		–
Smoking†	17 (14)	–		21 (20)	–		21 (23)	–		17 (17)	–		38 (7)
**Alcohol *n (%)***	–	>0.001		–	>0.001		–	>0.001		–	>0.001		–
Non-drinker	61 (51)	–		61 (58)	–		45 (49)	–		49 (49)	–		322 (56)
Drinker	38 (32)	–		32 (30)	–		30 (33)	–		38 (38)	–		246 (43)
Every-day drinker	20 (17)	–		12 (11)	–		17 (18)	–		13 (13)	–		3 (1)
**Civil state *n (%)***	–	0.041		–	0.010		–	>0.001		–	0.015		–
Single	64 (54)	–		59 (56)	–		44 (48)	–		59 (59)	–		298 (53)
Married	38 (32)	–		34 (32)	–		33 (36)	–		29 (29)	–		224 (40)
Divorced	6 (5)	–		7 (7)	–		8 (9)	–		6 (6)	–		9 (2)
Widow/widower	11 (9)	–		5 (5)	–		7 (8)	–		6 (6)	–		35 (6)

† Smoking or stopped smoking within the last six months.

* Difference between cases and controls, *p*-value for cases at each time point compared with controls.

‡ Variables with missing data. Education: two patients at inclusion, two patients at two months, one patient at four months, one control; employment: three controls, one patient at two months; smoking: one control; civil state: five controls.

### PD and risk of having CMD

Mean scores and prevalence rates were significantly higher for TB patients at inclusion compared with controls, for both SCL-8 and K-10 (Table 2). For both scales, a decrease in mean score and prevalence rates during treatment was observed.

**Table 2.** Mean scores and unadjusted prevalence rates of psychological distress estimated by SCL-8 and K-10

		SCL-8 (0–32)			Kessler 10 (10–50)				
			PD *n* (%)			PD *n* (%)			
	*N*	Mean (SD)	cut-off 1/0*		Mean (SD)	cut-off ≥17	cut-off ≥16	cut-off ≥15	cut-off ≥14
**Controls**	571	0.23 (0.26)	39 (6.8)		11.9 (2.1)	10 (1.8)	14 (2.5)	22 (3.9)	38 (6.7)
**TB cases**									
**At inclusion**	119	0.41 (0.33)	40 (33.6)		13.6 (2.8)	13 (10.9)	18 (15.1)	31 (26.1)	55 (46.2)
HIV-pos	21	0.46 (0.24)	6 (28.6)		14.1 (1.1)	1 (4.8)	2 (9.5)	7 (33.3)	14 (66.7)
HIV-neg	98	0.40 (0.35)	34 (34.7)		13.5 (3.1)	12 (12.2)	16 (16.3)	24 (24.5)	41 (41.8)
**At 2 months**	105	0.19 (0.21)	11 (10.5)		11.6 (1.5)	0 (0.0)	1 (1.0)	4 (3.8)	12 (11.4)
HIV-pos	17	0.28 (0.30)	4 (23.5)		11.9 (1.6)	0 (0.0)	0 (0.0)	0 (0.0)	4 (23.5)
HIV-neg	88	0.17 (0.18)	7 (8.0)		11.6 (1.5)	0 (0.0)	1 (1.1)	4 (4.6)	8 (9.1)
**At 4 months**	92	0.24 (0.30)	10 (10.9)		12.1 (2.5)	5 (5.4)	5 (5.4)	7 (7.6)	13 (14.1)
HIV-pos	19	0.32 (0.32)	3 (15.8)		12.6 (2.2)	2 (10.5)	2 (10.5)	3 (15.8)	3 (15.6)
HIV-neg	73	0.22 (0.30)	7 (9.6)		11.9 (2.5)	3 (4.11)	3 (4.11)	4 (5.5)	10 (13.7)
**At 6 months**	100	0.18 (0.27)	8 (8.0)		11.4 (1.6)	1 (1.0)	2 (2.0)	5 (5.0)	6 (6.0)
HIV-pos	23	0.22 (0.32)	1 (4.4)		11.7 (2.2)	1 (4.4)	1 (4.4)	1 (4.4)	2 (8.7)
HIV-neg	77	0.17 (0.25)	7 (9.1)		11.3 (1.4)	0 (0.0)	1 (1.3)	4 (5.2)	4 (5.2)

* Based on SCL-8 scale (item score 0–4); items dichotomised (SCL-8d), 0–1 categorised as negative (0) and 2–4 categorised as positive (1).

TB patients at inclusion had significantly greater OR of PD compared with controls, estimated by both SCL-8d and K-10; OR 6.91 (95% CI 4.19 to 11.39) and OR 12.05 (95% CI 7.40 to 19.63) (Table 3). After adjusting for potential confounders, the significant difference in prevalence of PD for TB patients at inclusion compared with controls remained; OR = 7.18 (95% CI 4.07 to 12.67) and OR = 14.52 (95% CI 8.15 to 25.84). At two months of follow-up, TB patients co-infected with HIV had a significantly greater OR of PD for both scales, whereas no significant difference was observed for non-co-infected patients (Table 3). At six months, no significant difference in PD prevalence rates was observed between all TB patients and controls. For both cases and controls, most prevalent symptoms for SCL-8 were feeling blue and feeling nervousness, and for K-10, feeling depressed and feeling tired (Table 4). Cronbach’s alpha was calculated to 0.711 for SCL-8d and 0.803 for K-10 (Table 4).

**Table 3.** Association between psychological distress and tuberculosis at each time point, crude and adjusted odds ratio (OR) by logistic regression

	PD defined by SCL-8d (cut-off 0/1)*			PD defined by K-10 (cut-off ≥14)	
	Crude OR (95 % CI) †	Adjusted OR (95 % CI) ‡, §		Crude OR (95 % CI)†	Adjusted OR (95 % CI) ‡, §
Controls	Ref.	Ref.		Ref.	Ref.
**TB cases**					
**At inclusion**	**6.91 (4.19–11.39)**	**7.18 (4.07–12.67)**		**12.05 (7.40–19.63)**	**14.52 (8.15–25.84)**
HIV-positive	**5.46 (2.01–14.85)**	**5.53 (1.87–16.31)**		**28.05 (10.69–73.64)**	**30.40 (10.68–86.49)**
HIV-negative	**7.25 (4.27–12.29)**	**7.89 (4.27–14.53)**		**10.09 (6.00–16.95)**	**11.84(6.37–22.03)**
**At 2 months**	1.60 (0.79–3.22)	1.77 (0.80–3.90)		1.81 (0.91–3.59)	1.98 (0.93–4.20)
HIV-positive	**4.20(1.31–13.48)**	**4.88(1.44–16.57)**		**4.32 (1.34–13.88)**	**4.66 (1.36–16.01)**
HIV-negative	1.18(0.51–2.72)	1.22 (0.46–3.27)		1.40 (0.63–3.11)	1.50 (0.61–3.66)
**At 4 months**	1.66 (0.80–3.46)	1.65 (0.72–3.77)		**2.31 (1.18–4.52)**	2.13 (0.99–4.63)
HIV-positive	2.56(0.71–9.16)	1.35 (0.31–5.85)		2.63 (0.73–9.42)	1.36 (0.32–5.75)
HIV-negative	1.45(0.62–3.37)	1.61 (0.62–4.20)		2.23 (0.06–4.68)	2.38 (1.01–5.65)
**At 6 months**	1.19 (0.54–2.62)	1.23 (0.51–2.96)		0.90 (0.37–2.18)	0.78 (0.29–2.06)
HIV-positive	0.62 (0.08–4.72)	0.41 (0.04–3.70)		1.34(0.30–5.91)	0.89 (0.18–4.33)
HIV-negative	1.36 (0.59–3.17)	1.50 (0.58–3.87)		0.77 (0.27–2.22)	0.65 (0.20–2.18)

* Based on SCL-8 scale (item score 0–4); items dichotomised, 0–1 categorised as negative (0) and 2–4 categorised as positive.

† Numbers available for crude analyses: 571 controls, 119 cases at inclusion, 105 cases at 2 months, 92 cases at 4 months and 100 cases at 6 months.

‡ Numbers available for adjusted analyses: 561 controls, 117 cases at inclusion, 103 cases at 2 months, 90 cases at 4 months and 100 cases at 6 months.

§ Adjusted for sex, age, and alcohol consumption.

**Table 4.** Prevalence of symptoms for controls and for TB patients at each time point

	TB Cases						
	At inclusion *N* (%)	At 2 months *N* (%)	At 4 months *N* (%)	At 6 months *N* (%)		Controls *N* (%)	Cronbach’s Alpha
SCL-8*							**0.711**
Nervousness and shakiness inside	78 (64.5)	37 (34.9)	34 (37.0)	29 (29.0)		243 (42.6)	0.667
Worrying too much about things	38 (31.4)	15 (14.2)	20 (21.7)	17 (17.0)		113 (19.8)	0.716
Feeling fearful	31 (25.6)	9 (8.5)	24 (26.1)	11 (11.0)		88 (15.4)	0.655
Feeling hopeless about the future	3 (2.5)	1 (0.9)	0 (0)	2 (2.0)		11 (1.9)	0.727
Feeling everything is an effort	40 (33.1)	13 (12.3)	11 (12.0)	15 (15.0)		61 (10.7)	0.670
Spells of terror or panic	18 (14.9)	9 (8.5)	10 (10.9)	8 (8.0)		35 (6.1)	0.666
Feeling Blue	91 (75.2)	54 (50.9)	50 (54.4)	41 (41.0)		383 (67.1)	0.687
Feelings of worthlessness	6 (5.0)	1 (0.9)	2 (2.2)	0 (0)		9 (1.6)	0.666
**Kessler 10**†							**0.803**
Feeling tired	94 (77.7)	56 (52.8)	51 (55.4)	43 (43.0)		302 (52.9)	0.795
Feeling nervous	75 (62.0)	36 (34.0)	34 (37.0)	28 (28.0)		223 (39.1)	0.787
Feeling so nervous that nothing could calm you down	4 (3.3)	0 (0)	0 (0)	0 (0)		7 (1.2)	0.775
Feeling hopelessness	7 (1.2)	1 (0.9)	0 (0)	0 (0)		7 (1.2)	0.785
Feeling restless of fidgety	9 (7.4)	0 (0)	5 (5.4)	0 (0)		14 (2.5)	0.772
Feeling so restless that you could not sit still	1 (0.8)	1 (0.9)	1 (1.1)	2 (2.0)		3 (0.5)	0.790
Feeling depressed	97 (80.2)	56 (52.8)	53 (57.6)	43 (43.0)		385 (67.4)	0.798
Feeling that everything is an effort	4 (3.3)	0 (0)	0 (0)	1 (1.0)		14 (2.5)	0.778
Feeling so sad that nothing could cheer you up	10 (8.3)	2 (1.9)	5 (5.4)	3 (3.0)		19 (3.3)	0.779
Feeling worthless	0 (0)	1 (0.9)	1 (1.1)	1 (1.0)		6 (1.1)	0.784

* Presence of symptoms is defined as item score >0, item scale 0–4.

† Presence of symptoms is defined as item score >1, item scale 1–5.

There were no significant differences between TB patients available and non-available to follow-up in demographic background variables and PD score at baseline (Data not shown).

## Discussion

### The association between CMD and TB

We observed a significantly higher prevalence of PD and risk of CMD among TB patients at the time of diagnosis, compared with the background population. Only one African study, placed in Nigeria, has compared CMD prevalence between newly diagnosed TB patients and healthy controls and found CMD among 30% of TB patients and 5% of the controls (Aghanwa and Erhabor, 1998). A published study based on a large dataset from the World Health Survey from 48 LMIC, defining TB patients from self-reported symptoms (Koyanagi *et al*., 2017), and a Korean study using a nationwide database, found comparable results (Oh *et al*., 2017). PD score declined during TB treatment, and no significant difference was observed after six months, which is consistent with observations from TB patients in Peru and TB co-infected HIV patients in Ethiopia (Ugarte-Gil *et al*., 2013; Deribew *et al*., 2013). Our observations of a decrease in PD score during treatment may indicate that active TB at diagnosis also displays symptoms of PD, which decrease upon treatment; however, it may also illustrate a decline in PD symptoms following improvement of TB.

The evidence of an association between CMD and TB is growing (Hayward *et al*., 2022), and a synergistic effect between CMD and TB has been suggested (Sweetland *et al*., 2017). CMD may cause delayed healthcare seeking, compromised immune status and impaired compliance to TB treatment, leading to increased community transmission, drug resistance and mortality, whereas TB may be a risk factor for CMD due to stigmatisation and economic consequences such as inability to work (Sweetland *et al*., 2017). Thus, screening and handling CMD in TB patients may be the next step to get on track with the goal of TB reduction.

### CMD prevalence and different scales and cut-offs

Low-resource settings carry both the burden of a high prevalence of TB and a substantial gap between prevalence of CMD and access to mental health care (Mental Health Atlas, 2020; WHO 2021>). To be able to find and treat CMD among TB patients, reliable CMD screening tools are needed. A cut-off at ≥16 suggested as lowest cut-off for K-10, by a validation study performed in a similar setting (Andersen *et al*., 2011), resulted in very low prevalence rates of PD in our population. SCL-8 was developed specifically for use in patients with physical disease, and hence, common somatic symptoms such as fatigue are not included in the scale. SCL-8d has not been validated in a low-resource setting but may be a useful CMD screening tool among TB patients that present with symptoms such as fatigue and weight loss. Our study underlines that there may be difficulties when using translated/adapted versions of screening tools, which are consistent with results from reviews on measuring depression and anxiety in sub-Saharan Africa (Sweetland *et al*., 2014), and the clinical use of the Kessler scales (Stolk *et al*., 2014).

## Strengths and limitations

Strengths of our study include standardised diagnosis, treatment and follow-up of TB patients. We had the possibility of obtaining a randomised sample from the background population and to reproduce uniform interviews for both PD scales with no inter-individual variance, as all interviews were performed by only one field assistant. Screening for PD among controls has been performed parallel with the screening of PD among cases, thus potential season variability is considered as low. Our study is one of the few studies that have investigated the difference in PD prevalence between TB patients and the background population in Africa. and we contribute with the first data from Guinea-Bissau on PD in the background population. Our findings of PD and risk of CMD prevalence rates are within the realistic end of ranges compared with previously published studies from Africa, where CMD prevalence rates range from 3.1% to 77.7% among the background population and from 28 to 80% among TB patients (Issa *et al*., 2009; Peltzer *et al*., 2012; Steel *et al*., 2014; Hayward *et al*., 2022). Internal consistency was acceptable for SCL-8d with Cronbach’s alpha 0.711 and good for K-10 with Cronbach’s alpha 0.820.

Our study has a number of limitations. Firstly, the number of controls eligible for CMD screening was lower than we expected and the distribution of sex was unequal between TB cases and controls. However, it is well known that TB is more prevalent among males and CMD more prevalent among females (Neyrolles and Quintana-Murci, 2009; Steel *et al*., 2014), and the unequal sex distribution would cause a reduction of the association between TB and PD in our sample. This is consistent with our observations of an even higher OR of PD for TB patients, after adjusting for sex and other possible confounders.

Secondly, we were not able to collect follow-up data for each TB patient. Significant difference in background characteristics compared with the background population varied between TB patients at each time point. We investigated if TB patients with PD at baseline were less likely to show up to clinical controls and if their absence cause a reduction in mean PD score at follow-up. However, no such difference was observed. Furthermore, as we had a complete dataset at inclusion, our main finding of a clear association of TB and PD at the time of diagnosis was not influenced by the loss to follow-up.

Thirdly, although controls were screened for TB, there may be controls with TB in our cohort (Porskrog *et al*., 2011). Yet, this would cause a reduction in the association between TB and PD in our sample and would not have weakened our main findings of an association between TB and PD at inclusion.

Fourthly, the study was limited by the lack of referral options for those screening out with mental disorders. This is a general ethical challenge in global mental health, and it is very suboptimal to uncover conditions for which the local standard of care is no care. Yet, we have seen within the field of HIV that describing the burden of disease may be a first step towards changing this situation and opening pathways for future care options.

### Public health importance and future directions

Screening for and treating CMD in TB patients may be an important element in strategies to reduce the burden of TB. Further studies are needed to find reliable CMD screening tools, easy to use for trained healthcare providers, and to explore CMD treatment options and interventions among TB patients in LMIC.

## Conclusion

Screening for psychological distress revealed a significantly higher prevalence of risk of common mental disorder among TB patients at the time of diagnosis compared with the background population, with a decrease in prevalence rates among TB patients during treatment.
